# Where Epigenetics Meets Food Intake: Their Interaction in the Development/Severity of Gout and Therapeutic Perspectives

**DOI:** 10.3389/fimmu.2021.752359

**Published:** 2021-09-17

**Authors:** Philippe T. Georgel, Philippe Georgel

**Affiliations:** ^1^Department of Biological Sciences, Cell Differentiation and Development Center, Joan C. Edwards School of Medicine, Byrd Biotechnology Science Center, Marshall University, Huntington, WV, United States; ^2^Laboratoire d’ImmunoRhumatologie Moléculaire, Institut National de la Santé et de la Recherche Médicale (INSERM) UMR_S 1109, Institut thématique interdisciplinaire (ITI) de Médecine de Précision de Strasbourg, Transplantex NG, Faculté de Médecine, Fédération Hospitalo-Universitaire OMICARE, Fédération de Médecine Translationnelle de Strasbourg (FMTS), Université de Strasbourg, Strasbourg, France; ^3^Unité de Recherche et d'Expertise Immunity and Inflammation, Institut Pasteur in New Caledonia, Pasteur Network, Nouméa, New Caledonia

**Keywords:** gout, hyperuricemia, food intake, epigenetics, genetic variants, trained immunity 2

## Abstract

Gout is the most frequent form of inflammatory arthritis in the world. Its prevalence is particularly elevated in specific geographical areas such as in the Oceania/Pacific region and is rising in the US, Europe, and Asia. Gout is a severe and painful disease, in which co-morbidities are responsible for a significant reduction in life expectancy. However, gout patients remain ostracized because the disease is still considered “self-inflicted”, as a result of unhealthy lifestyle and excessive food and alcohol intake. While the etiology of gout flares is clearly associated with the presence of monosodium urate (MSU) crystal deposits, several major questions remain unanswered, such as the relationships between diet, hyperuricemia and gout flares or the mechanisms by which urate induces inflammation. Recent advances have identified gene variants associated with gout incidence. Nevertheless, genetic origins of gout combined to diet-related possible uric acid overproduction account for the symptoms in only a minor portion of patients. Hence, additional factors must be at play. Here, we review the impact of epigenetic mechanisms in which nutrients (such as ω-3 polyunsaturated fatty acids) and/or dietary-derived metabolites (like urate) trigger anti/pro-inflammatory responses that may participate in gout pathogenesis and severity. We propose that simple dietary regimens may be beneficial to complement therapeutic management or contribute to the prevention of flares in gout patients.

## Introduction

Gout is probably the oldest known joint disease, already described by Hippocrates of Kos in the fifth century B.C (1). Gout has been dubbed the “disease of Kings” and indeed, considering only the French royalty, 20 of the 34 kings of France were said to have been afflicted by it (2). Since then, gout has been associated with a decadent and unhealthy lifestyle, caused by excessive calorie-rich food intake. Unfortunately, such considerations are still prevalent, and gout continues to be considered a self-inflicted disease. Importantly, a recent meta-analysis (3) revealed that, in contrast to this common belief, diet actually only explained some of the variation(s) in serum urate levels, compared to the importance of the genetic contribution. Such data have important psychological consequences, as they should help to relieve the stigmatization of gout patients and encourage them to seek medical help during the first crisis, improve their therapeutic observance, limit the duration of their therapy, and diminish the social and economic burden of the disease. Although this meta-analysis concerned patients of European ancestry and may not be generalized to other ethnic groups (specifically those, like Oceanians (4) in which gout represents a major public healthcare issue), it highlights the importance of genetic variants identified in genome-wide association studies (GWAS), which account for almost one fourth (23.9%) of the variation of serum urate levels compared to 0.3% associated with diet. This study does not however explain what is responsible for the majority of the variation(s) of serum urate levels (approximately 75%). This indicates that, even though hyperuricemia and the subsequent formation of urate crystals have been known for a long time as major determinants of gout etiology, many facets of this disease, which remains the most prevalent form of inflammatory arthritis worldwide, are still unexplained.

Epigenetics describes the mechanisms by which changes in genes expression occur independently from the primary DNA sequence (genome) of an organism. They are divided into three main categories: (i) changes in DNA methylation affecting chromatin compaction, (ii) chromatin alterations through histone post-translational modifications (PTM) and/or incorporation of histone variants and (iii) regulation by non-coding RNAs (ncRNAs), such as microRNAs (miRs) or long non-coding RNAs (lncRNAs) (5). Such modifications are considered as important responses to environmental triggers, enabling adaptive changes of gene expression patterns (6). In the context of gout pathogenesis, epigenetic mechanisms might be of particular interest to account for the remarkable variability seen in patients (7) in terms of crisis intensity or frequency and to assess the relationship [or sometimes the absence of relationship (8)] between uricemia levels and MSU crystals-dependent joint inflammation. In line with these assumptions, it is noteworthy to highlight that genome-wide association studies (GWAS) comparing gout patients to control groups have identified variants in genes encoding important players in epigenetic regulation mechanisms, such as DNA Methyl Transferase 1 (DNMT1) (9) and lncRNAs (10). Furthermore, and as noted above, food intake, despite the recent re-evaluation of its importance in gout development, remains an important environmental source of precursors of uric acid, a molecule that has been shown to affect the physiological and inflammatory properties of monocytes through epigenetic-driven reprogramming (11). This process, coined “trained innate immunity”, appears nowadays as a major player in the defense against pathogens and in autoimmunity (12). Finally, besides providing urate precursors, diet is also a source of short-chain fatty acids (which can also originate from the microbiota) that are known to induce epigenetic changes through, most commonly, changes in histone acetylation, and consequently affect pathophysiological responses (13), as shown using a gout mouse model (14).

Up to now, the therapeutic management of gout patients aims, in the short term, at treating the gout flares, and in the long term, at reducing urate levels (15). For these purposes, several molecules are available, among which are colchicine, IL-1β blockers, non-steroidal anti-inflammatory drugs (NSAID), and drugs facilitating urate renal excretion (like Probenecid) or decreasing its production (such as the Xanthine Oxidase inhibitors Allopurinol and Febuxostat). Unfortunately, these treatments can generate serious side effects, including renal and cardiac damages or hepatotoxicity (16). Further, these drugs focus on diminishing the symptoms rather than curing the patient. Of note, novel molecules targeting the aggregation of neutrophil extracellular traps are in development (17). In the long term, though with limited expected benefits, lifestyle modifications are also recommended (15). The possibility that epigenetic mechanisms may contribute to gout pathogenesis offers the potential of a set of completely different therapeutic options and, more importantly, lasting management of patients through epigenetic reprogramming of innate immune cells such as monocytes/macrophages (for instance with the Histone DeAcetylase -HDAC - inhibitor Vorinostat), which are the main producers of Il-1β, a crucial cytokine in gout pathogenesis. This strategy has been already considered for other rheumatic diseases, such as rheumatoid arthritis or systemic lupus erythematosus (18), and implemented in animal models (19). Diet counseling could also be adapted, as indicated by recent findings linking salt (20) or other components (such as hydrogen sulfide) (21) and inflammation as a result of innate immune cells reprogramming (22).

Here, we review the current mechanistic understanding of how several components provided by food intake are capable of modulating inflammation through epigenetic modification/reprogramming of innate cells and how that knowledge could be translated into actionable decisions for the benefit of gout patients.

## Gout and Food Intake: Not Only Urate, Not Only Inflammasome-Dependent

**Figure 1:**
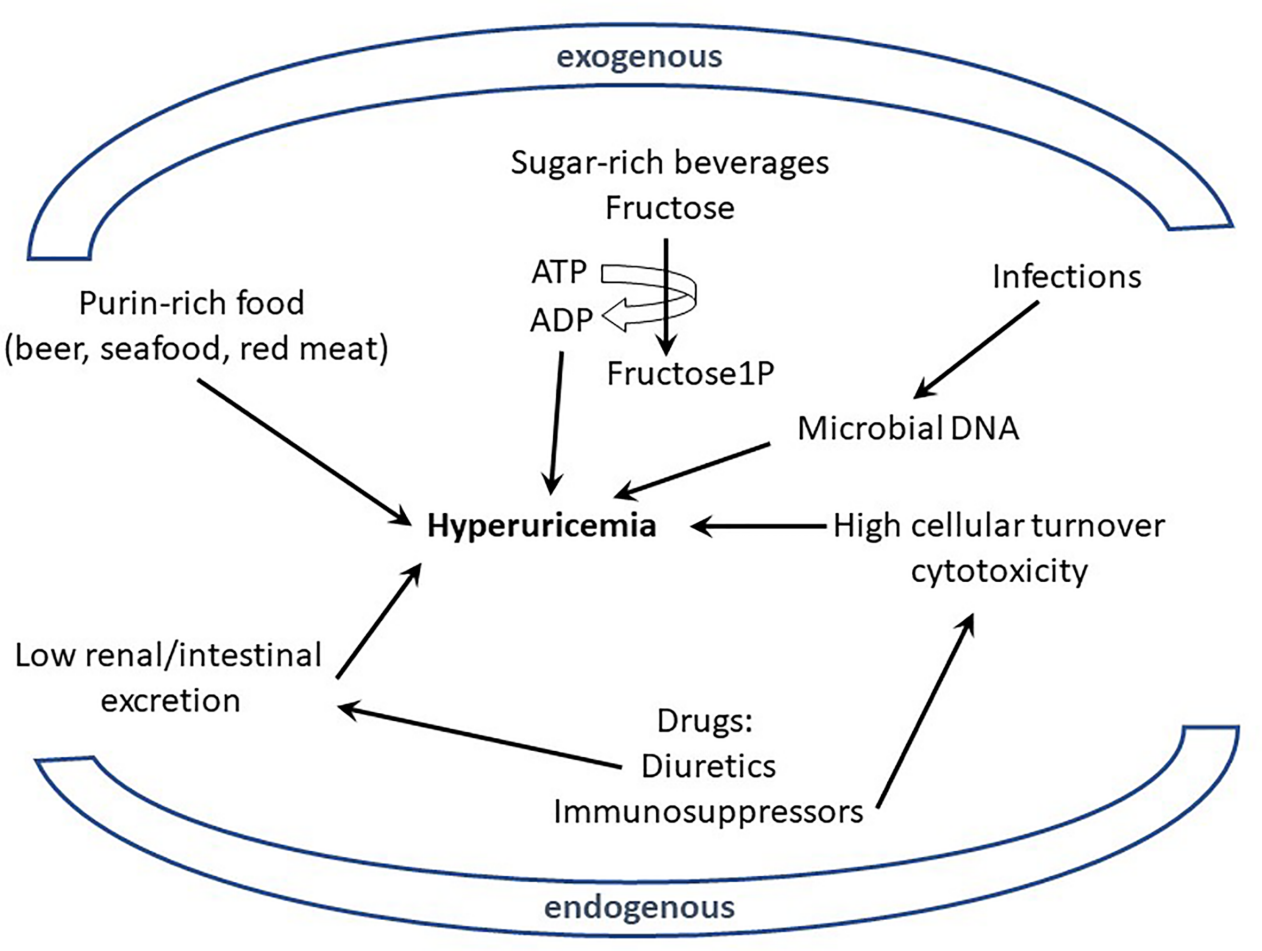
Excessive uric acid in the blood (hyperuricemia) can originate from endogenous or exogenous sources. Various nutrients (purin-rich such as red meat or sugar-rich beverages) have been shown to be environmental (exogenous) factors participating in hyperuricemia; similarly intrinsic (endogenous) determinants from the host, like genetic defects affecting urate excretion, can also participate to hyperuricemia. Metabolism of elevated DNA concentration resulting from infections (exogenous) or cell cytotoxicity (endogenous) is also suspected to generate high levels of urate.

Excessive uric acid in the blood circulation (hyperuricemia) and the subsequent formation and deposition of MSU crystals have been known for more than a century [with the work of Sir Alfred Garrod (2)] to be the etiological trigger of gout (23). However, the connections between elevated urate, MSU, and gout flares are more complex than a simple cause and effect relationship. Indeed, only 2-15% of hyperuricemic individuals develop clinical gout and conversely, some patients exhibit urate levels within normal range at the time when they suffer acute gout flares (23). Nevertheless, the impact of MSU crystals on inflammation and pain is widely recognized. Of note, the loss of URICASE activity in Humans and other Primates explains high urate levels in these species (23). The mutations inactivating the *URICASE* gene that have been selected suggest that urate also carries some beneficial effects, including antioxidant and adjuvant properties, which protect the host against neurodegenerative or infectious diseases (24). As mentioned above, diet has always been associated with gout flares, but this assumption has been recently objectified in a meta-analysis regrouping 16,760 individuals (3). This work demonstrated that the consumption of specific nutrients (beer, liquor, wine, potato, poultry, soft drinks, and meat) increased serum urate levels, while others (eggs, cheese, peanuts) were associated with lower levels. These data are in line with observations linking Mediterranean diet (in which red meat intake is moderate) with reduced gout incidence (25). However, the mechanisms by which nutrition modifies urate production and impacts on gout crises remain elusive. Indeed, urate, the end-product of purine metabolism, can have endogenous or exogenous origins and it is widely accepted that purines originate equally from diet and synthesis under normal circumstances (26). Hyperuricemia can therefore result from two kinds of perturbations: (i) those resulting from excessive purine-rich food, infections, or neoplasms and (ii) those resulting from reduced excretion of urate (**Figure 1**).

With regards to the exogenous origin of hyperuricemia, several nutrients, such as red meat (rich in purines) and fructose-rich beverages appear as clear contributors. Of note, fructose metabolism in the liver, through unregulated phosphorylation of adenosine triphosphate into adenosine monophosphate, increases urate production (27). However, such direct connection is more difficult to validate when one considers that others purine-rich foods of vegetable origin (bean, lentils, peas…) are not known to contribute to any increased risk of hyperuricemia and gout flares (28). Such contradiction indicates that other dietary components, besides those which drive purine-derived uric acid overproduction, are involved in gout pathogenesis.

Indeed, high protein diet-induced gout in chicken has recently been described in a recent report (29). While this regimen also induced serum urate levels, another study in human’s suffering hypertension (30) provided contradictory insights by showing lower serum urate levels in patients (mean BMI 30.2) which received a protein-rich diet for 6 weeks. Such contrasting data might reflect differences between chicken and human metabolism, or alternatively between the composition and the origin of the proteins that were enriched in these dietary regimens.

Comparatively to that of proteins and sugar, the impact of lipids on the ignition of gout flares or the severity of the symptoms has seldom been investigated, which is surprising given the strong association between this disease and obesity (31, 32). This issue has been recently explored in a gout mouse model following footpad injections of MSU crystals in animals which were simultaneously fed with a high fat diet (containing 10% yeast extracts, likely to be an ω-6 Fatty acid-rich diet, low in ω-3 Fatty acids) (33). Interestingly, this model reproduced the rise in inflammatory cytokines seen in patients during acute gout flares, as well as the intestinal dysbiosis (commonly associated with high PUFA diet) which is also a hallmark of the disease (34). In another study, the authors analyzed the impact of a fiber-rich food on gout induced by intra-articular injections of MSU crystals in mice (35). Indeed, consumption of fibers, which are abundant in the Mediterranean diet, was shown to limit gout symptoms in humans. Of note, this study reported a faster resolution of the MSU crystal-induced inflammation in high fiber diet-fed animals. Mechanistically, this effect appears to be mediated by short chain fatty acids (SCFA) such as acetate which are produced upon fibers digestion. Of note, an increase of free acetyl Co-A has been shown to contribute to global and local histone hyper-acetylation (to be discussed in sections C and D). Remarkably, providing acetate in the drinking water of mice reduced inflammatory responses, even after MSU crystals injection. Overall, this work suggests that food processing by the intestinal flora can generate lipid compounds, such as acetate, which exhibit a beneficial action (anti-inflammatory) on immune cells. Other lipids, like Omega-3 polyunsaturated fatty acids (ω-3 PUFA), are considered potentially beneficial in gouty arthritis patients. This assumption relies on investigations in gout patients in which questionnaires enabled to observe that the regular consumption of ω-3 PUFA-rich fish was associated to reduced risk of gout flares (36). Of note, taking ω-3 PUFA alone (as a dietary supplement) seemed to have no impact, but the limited number of participants who reported to use such supplements did not enable the authors to be conclusive. Importantly, serum urate levels were not quantified in this study; it is therefore possible to explain the beneficial effect of ω-3 PUFA-rich fish diet by concomitant reduced red meat consumption, anti-inflammatory properties of this compound, or other mechanisms. Indeed, this family of molecules was shown to inhibit the activation of the Nlrp3 (NOD-like receptor family, pyrin domain containing 3) inflammasome (see below) in murine bone marrow-derived macrophages, an effect that could very well account for their anti-inflammatory properties and efficacy against gout, a disease in which the NLRP3 inflammasome has been strongly implicated (37, 38).

Altogether, it appears that the contribution of diet in the development/severity of gout is far more complex than previously anticipated and cannot be simply reduced to a purin rich/poor dichotomic classification. This likely contributes to the poor impact and optimization of dietary education and counseling in the management of gout patients (39, 40).

Finally, in addition to this information showing that diet-derived urate production appears, at best as a contributing factor to gout pathogenesis, it must also be emphasized that the mechanisms linking MSU crystals and severe inflammation remain partially uncovered. While association between the presence of these crystals in the joints and the incidence of gout flares is undisputable (28), several questions are still unanswered. For instance, the mechanisms by which MSU crystals induce an inflammatory response awaits definitive answers. While it has been known for several years that the NLRP3 inflammasome plays a crucial role in this process (37), the model of its activation has been described in cells (mostly macrophages) in which a “priming signal” is delivered by Lipopolysaccharide (LPS) addition, prior to the “activation signal” provided by MSU crystals. *In vivo*, however, MSU crystals alone can induce an inflammatory response manifested by IL-1β secretion and the nature of the priming signal in these conditions remains to be identified. In addition, several groups reported that injection of MSU crystals is able to promote inflammation, even in the absence of the Nlrp3 inflammasome, at least in mouse models (41–43).

All these gaps in knowledge about the relationships between diet, urate production and inflammatory responses prompted us to consider alternative (but not exclusive) explanations to account for their respective involvement in gout pathogenesis. In the next sections, we are attempting to develop an alternative (and under-explored) hypothetical model based on evidences linking epigenetics and trained immunity as critical determinants playing an important role in the modulation of inflammatory responses in the presence of MSU crystals and depending on dietary components.

## Genetics and Epigenetics of Gout

Many GWAS performed to identify variants involved in hyperuricemia revealed the importance of genes (such as *SL2A9* or *ABCG2*) regulating the metabolism/transport/excretion of urate. Some of these genes/variants were also associated in gout patients *vs* controls GWAS studies, which, in addition, revealed the importance of genes (like *IL-1β* or *TLR*4) participating in inflammatory pathways [reviewed in (44, 45)]. These observations had important therapeutic ramifications, as they permitted consideration of gout not only as a metabolic, but also as an auto-inflammatory disease. These studies also demonstrated that although monogenic disorders can be associated to gout, its genetic origin is usually complex, involving multiple genomic loci. Importantly, heritability of urate levels (performed in twin studies) was estimated to average 50%, while that of gout appeared variable, from very low (46) to 30% (47). Some of the genes identified by this approach offer opportunities to develop important therapeutic improvement and enable precision medicine, for instance in gout patients carrying a loss-of-function allele of the main urate transporter gene *SLC22A12* (48, 49) and in which uricosuric agents, like probenecid, are inefficient. However, genetic defects still cannot be identified as the main trigger of the disease for many patients, since known sequence variants explain less than 10% of the risk of developing gout (50). This leaves room for additional mechanisms accounting for gout pathogenesis and eventually ensuing progression from hyperuricemia to gout flares. Interestingly, gout-related polymorphisms were recently identified in genes encoding epigenetic players which are known to modulate immune cell activity (51), opening novel avenues of research and unsuspected mechanistic insights in gout pathogenesis.

Epigenetic-related genes involved in gout pathogenesis encode microRNAs, such as miR-302F, which was identified in GWAS studies (52) or miR-221-5p, which is differentially expressed in the serum of gout patients compared to controls (53). Importantly, this miRNA can modulate *IL-1β* expression, as evidenced by a luciferase assay (53). Long Intergenic Non-Coding RNAs (LINC) were also associated with gout in various studies [reviewed in (10)]. MicroRNAs and long non-coding RNAs are transcripts which are not translated into proteins, but affect the expression of multiples genes through various mechanisms (translational inhibition, enhancement of mRNA degradation). While the identification of the genes that are targeted by non-coding RNAs remains challenging *in vivo*, the stability of these molecules and the possibility to easily quantify them in body fluids makes non-coding RNAs attractive circulating biomarkers (54). Additionally, the role of DNA methylation in gout was demonstrated through the discovery of a variant (rs2228611) in the *DNA MethylTransferase 1* (*DNMT1*) gene whose presence is statistically increased in gout patients compared to controls (9). In line with this information, an innovative multi-OMICs study, in which the cell-specific methylome was compared between gout patients and controls, revealed that many differentially methylated gene regulatory genomic sites were associated with *IL-1β* expression in monocytes (55).

**Figure 2:**
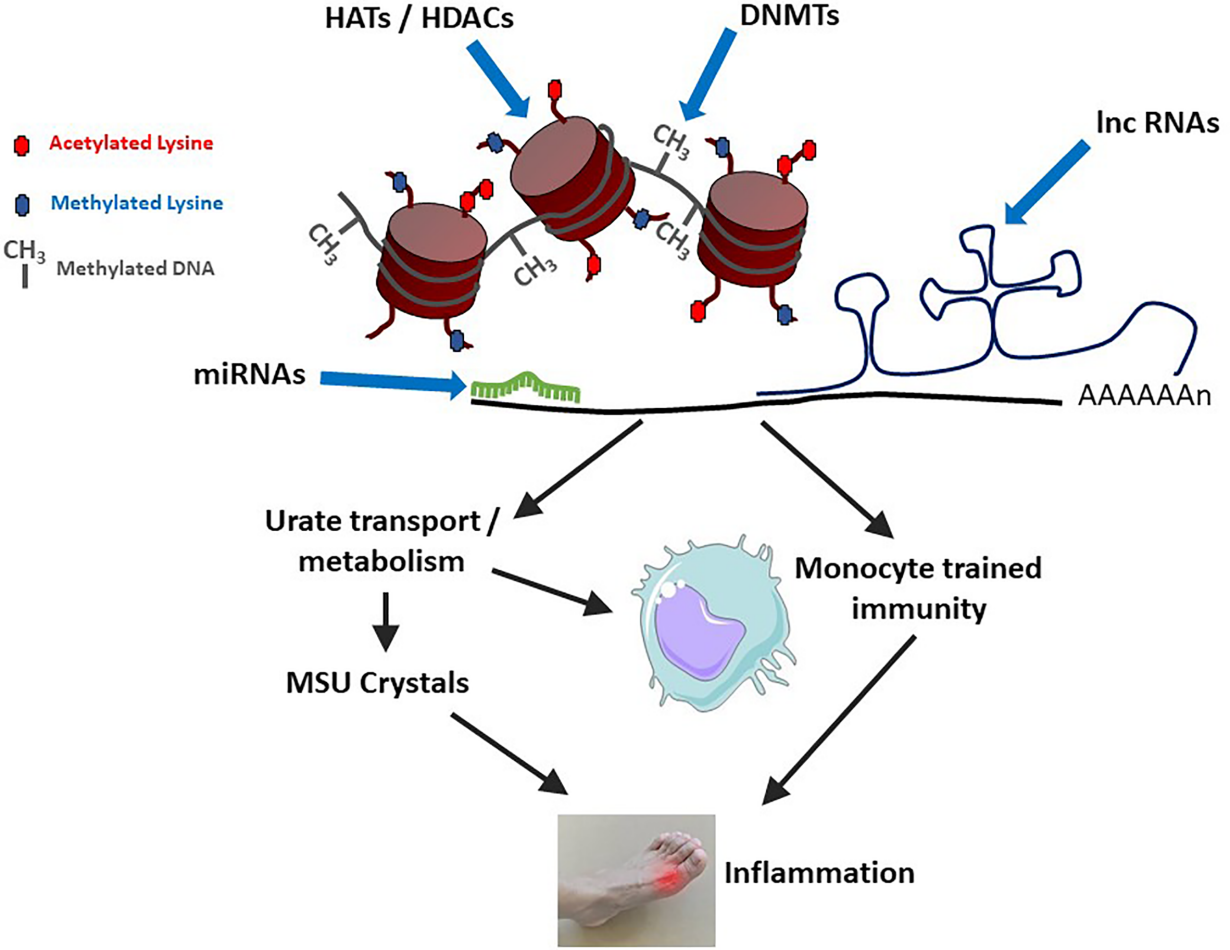
Epigenetic mechanisms modulate gouty inflammation. DNA and histone modifications (by DNA Methyl Transferases – DNMTs or Histone Acetyl Transferases – HATs/Histone Deacetylases - HDACs) affect DNA compaction and subsequent gene transcription. At the mRNA level, gene expression is modulated by microRNAs (miRNAs) and long non-coding RNAs (lnc RNAs). At least two categories of gout-related genes can be affected: (i) those directly regulating urate metabolism/excretion and (ii) those affecting macrophages polarization and activation status. Increased levels of circulating uric acid, in combination with a pro-M1 macrophage polarization (which can also be favorized by high urate conditions) can lead to monosodium urate (MSU) crystals formation and concomitant inflammation.

Epigenetic mechanisms can affect gout physiopathology through various ways, like changes in gene expression resulting from modifications of DNA methylation levels or histone post-translational modifications, including acetylation/deacetylations and methylation/demethylation processes. Such genes may encode proteins regulating urate metabolism/excretion. More recently, epigenetic-driven modifications of gene expression were also shown to sustain innate immune memory, a feature of innate immune cells which is also termed “trained immunity” [reviewed in (56)] and that occurs following cell stimulation with Pathogen- or Danger-Associated Molecular Patterns (PAMPs, DAMPs) such as β-glucans (57). While the impact of urate on histone methylation and *IL-1Ra* inhibition was described several years ago (58), the concept of innate immune reprogramming has gained considerable interest and has now major consequences for inflammatory diseases, including gout (11). These different mechanisms are schematized in **Figure 2**.

## Diet-Derived Epigenetic Modifiers

**Figure 3:**
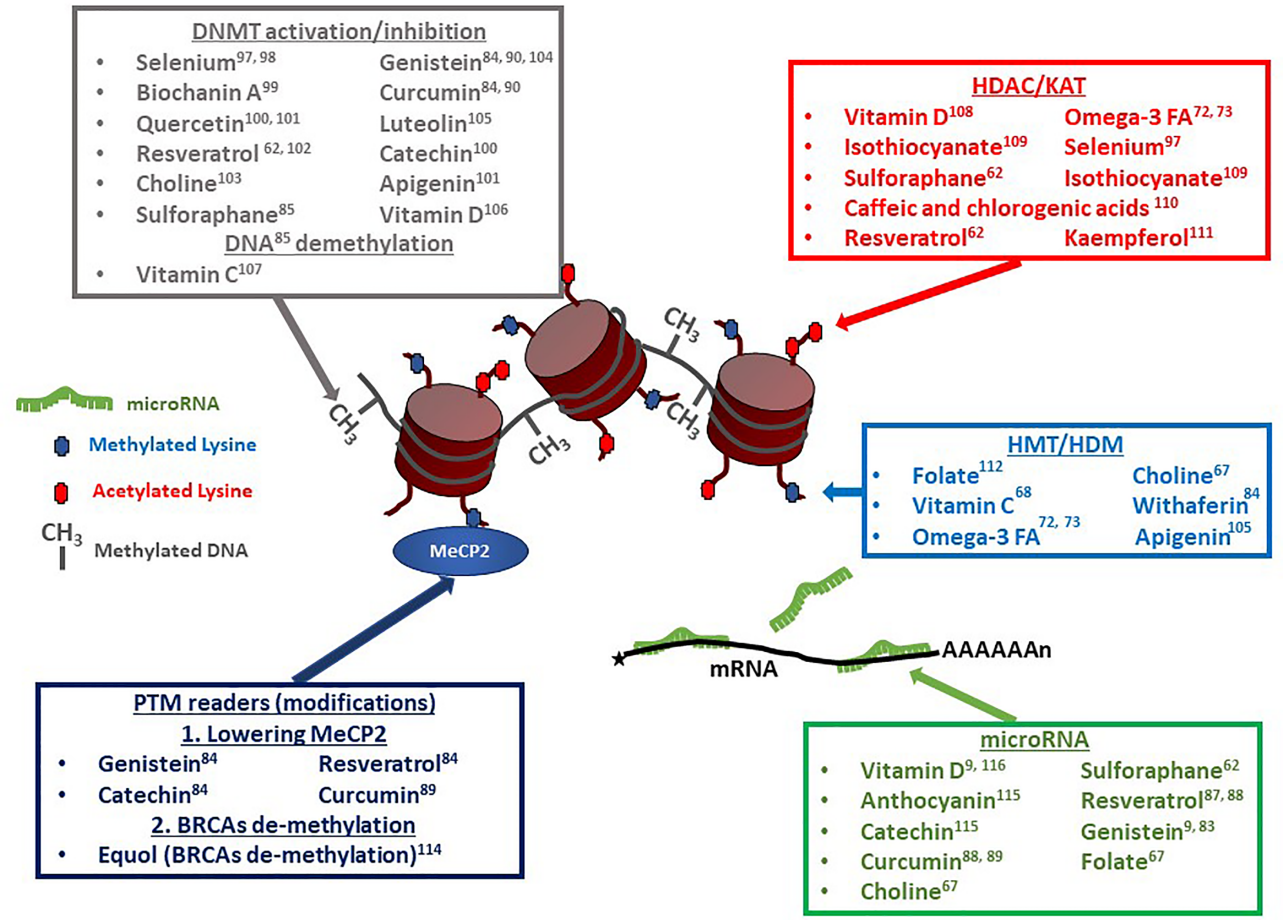
Dietary compounds affecting epigenetic regulatory events. Multiple compounds have pleiotropic effects on chromatin modifiers, transcription factors, and microRNAs (miRs). Vitamin D, Resveratrol and Sulforaphane, for example, can affect DNA methylation levels, as well as histone acetylation and microRNA levels. Resveratrol can additionally affect the transcription factor MeCP2 ‘s expression levels. DNMT, DNA-Methyl Transferase (60–64, 66, 67, 69, 71–73, 75, 91–93); HDAC, Histone De-acetylase (74, 77–80, 88, 91, 92, 94); KAT, Lysine Acetyl Transferase; HMT, Histone Methyl Transferase (67, 72, 77, 81, 84, 95, 96); HDM, Histone De-Methylase; PTM, Post-Translational Modification; MeCP2, Methyl CpG Binding Protein 2 (67); BRCA, BRCA1 DNA Repair-Associated protein (86); miR, microRNA (9, 68, 70, 81, 83, 85, 87, 91, 97, 98).

Because epigenetics has now been identified as an important player in gouty arthritis, we thought to re-evaluate the impact of diet in light of nutrient-derived epigenetic modifiers. As described in the previous section, a significant number of epigenetic factors potentially affect gout onset and can contribute to further inflammation events worsening the symptoms (9, 55). Among such factors influencing epigenetic changes are multiple compounds (listed in **Table 1**) that are commonly found in one’s diet. A recent review by Li and colleagues (90) describes the multiple epigenetic effects engendered by bioactive dietary compounds (summarized in **Figure 3**). DNA methylation (9, 53), as well as changes in histone post-translational modifications (99), and microRNA and long non-coding RNA can be modulated by dietary input and play a role in reducing inflammation (100). A subset of diet-derived epigenetic modifiers has already been identified as modulators of risk of developing gout (65, 91). The meta-analysis performed by Li et al. (90) linked red meat, seafoods, alcohol, sweetened drinks, and dairy products to the list of factors increasing risks of gout and hyperuricemia.

**Table 1:** List of dietary-derived epigenetic modifiers classified according to their mode of action and corresponding references.

Epigenetic regulation	Food Ingredient	Reference
DNMT activation/Inhibition	Selenium	Xiang et al. (59)
		Davis et al. (60)
	Biochanin A	Ito et al. (61)
	Quercetin	Lee et al. (62)
		Fang et al. (63)
	Resveratrol	Kala et al. (64)
		Gao et al. (65)
	Choline	Wolff et al. (66)
	Sulforaphane	Li et al. (67)
	Genistein	Mirza et al. (68)
		Nagaraju et al. (69)
	Curcumin	Mirza et al. (68)
		Zheng et al. (70)
	Luteolin	Kanwal et al. (71)
	Catechin	Lee et al. (62)
		Kanwal et al. (71)
	Apigenin	Fang et al. (63)
	Vitamin D	Tapp et al. (72)
	Vitamin C	Young et al. (73)
HDAC/KAT	Sulforaphane	Gao et al. (65)
	Isothiocynanate	Beklemisheva et al. (74)
	Vitamin D	Fetahu et al. (75)
	Omega-3 FA	Patterson et al. (76)
		Abbas et al. (77)
	Selenium	Xiang et al. (59)
	Caffeic acid	Bora-Tatar et al. (78)
	Resveratrol	Gao et al. (65)
	Kampferol	Berger et al. (79)
HMT/HDM	Folate	Mentch & Locasale (80)
	Vitamin C	Yin et al. (81)
	Omega-3 FA	Abbas et al. (77)
	Choline	Pogribny et al. (82)
	Withaferin	Mirza et al. (68)
	Apigenin	Kanwal et al. (71)
PTM readers (MeCP2)	Genistein	Mirza et al. (68)
	Catechin	Mirza et al. (68)
	Resveratrol	Mirza et al. (68)
	Curcumin	Mizraei et al. (83)
PTM readers (BRCA)	Equol	Bosviel et al. (84)
MicroRNA	Vitamin D	Nunez-Lopez et al. (9)
		Fan et al. (85)
	Anthocyanin	Arola-Arnal & Blade (86)
	Catechin	Arola-Arnal & Blade (86)
	Curcumin	Mizraei et al. (83)
		Xin et al. (87)
	Choline	Pogribny et al. (82)
	Sulforaphane	Gao et al. (65)
	Resveratrol	Xin et al. (87)
		Qin et al. (88)
	Genistein	Zhong et al. (9)
		Hirata et al. (89)
	Folate	Pogribny et al. (82)

DNMT, DNA-Methyl Transferase; HDAC, Histone De-acetylase; KAT, Lysine Acetyl Transferase; HMT, Histone Methyl Transferase; HDM, Histone De-Methylase; PTM, Post-Translational Modification; MeCP2, Methyl CpG Binding Protein 2; BRCA, BRCA1 DNA Repair-Associated protein; miR, microRNA.

In addition to these factors, specific DNA methylation or demethylation changes can be mediated by DNMT activation or inhibition [DNMT1 in particular, but also DNMT3A and DNMT3B (9)] induced by dietary compounds (**Figure 3**). Two specific dietary compounds have been identified as affecting gout’s severity by controlling changes in DNA methylation levels of genes inflammation (65). Deficiencies in folic acids in the diet results in dis-regulation of *IL-1β*, *IL-6* and *TNF-α* genes methylation patterns (101). Similarly, polyphenolic compounds, such as curcumin, genistein, or resveratrol, mediate changes in DNA methylation patterns (65). Curcumin, specifically plays a role in reducing activation of NF-κB, as well as affecting microRNA expression (102).

Methyl donors, in addition to potentially changing DNA methylation patterns are also involved in histone methylation/demethylation processes. For example, the inflammatory response associated with gout can be mediated by tri-methylation of histone H3 Lysine 4 (H3K4me3) (103). Similarly, urate-induced inflammation priming can be reduced by using the histone methyl transferase inhibitor (HMTI) 5’-deoxy-5’-methylthioadenosine (MTA), resulting in limited cytokine production (11, 82). Significant evidence has been gathered to indicate that dietary compounds affecting the DNA methylation level, such as choline (81), vitamin C (95), have also an effect on histone methylation levels and genomic distribution, suggesting a complex interplay between various epigenetic regulatory mechanisms.

The next major epigenetic regulatory component involves histone acetylation. Multiple studies have identified Histone De-Acetylase inhibitors (also referred to as HDACi) as drugs capable of reducing inflammation. Romidepsin, an HDACi for HDAC class1 and class 2, increases Suppressor Of Cytokine Signaling protein 1 (SOCS1) expression leading to a decrease in IL-1β (104). Treatment of peripheral blood mononuclear cells (PBMCs) with sodium butyrate, a less selective HDACi, results in decreased NF-κB expression, and helps suppressing MSU-induced inflammation through a reduction of *IL-6*, *IL-8* and *IL-1β* transcription in PBMCs (105). As was discussed in the preceding section, dietary compounds are also capable of regulating the level of histone acetylation. Vitamin D can reduce inflammation through its action on IL-17A (76). The polyphenolic Epigallocatechin-3 Gallate (EGCG) has been shown to prevent acute gout through its anti-inflammatory activity by suppressing the activation of the NLRP3 inflammasome, acting as a modulator of Lysine Acetyl Transferase (KAT) (50). Short Chains Fatty Acid (SCFA) and ω-3 FAs can also contribute to lowering IL-1β release and NLRP3-mediated caspase 1 activation. Diets using longer poly-unsaturated fatty acids (PUFA), such as docosahexaenoic acid (DHA) and eicosapentaenoic acid (EPA), have been shown to have beneficial effects on cancer cell lines and patients suffering of various types of cancer, through lowering inflammation [for review, see (77)], but perhaps most importantly in the context of this review, through a mechanism leading to a global hyper-acetylation of histone N-termini, as well as at specific loci (94). The high ω-3 FA diet, by inhibiting the enzyme acetyl-CoA carboxylase which function is to convert two acetyl-Co-A into a malonyl-Co-A, leads to an increase in the free pool of acetyl Co-A (which is the acetyl donor for histone acetylation) (106, 107). An unexpected secondary effect of diets rich in ω-3 FA is a modification in the expression level of several microRNAs related to gouty inflammation, specifically miR-146a and miR-155 (100). Note that the mode of action for these observed changes in miR levels is likely to be indirect and mediated by changes in chromatin accessibility through histone hyper-acetylation of the target genes’ regulatory elements. miR-155, when over-expressed, has been associated with an increased production of MSU-induced pro-inflammatory cytokines (100).

MicroRNAs expression levels have often been shown to be modulated by various types of dietary polyphenolic compounds. For example, curcumin, which was previously mentioned as affecting DNA methylation pattern (see previous section), is also involved in lowering inflammation (decreased NF-κB expression) through modifying the patterns of expression of miRs (65). Another aspect of the importance of miRs as part of the diet is suggested by the concept of «dietary xenomiRs», miRs coming directly from one’s diet (plants (108) or other origins) and used by the consumer’s cells (109). It indicates that not only do dietary compounds influence the miR expression in the host cells, but the xenomiRs themselves can provide an additional level of complexity to the effects of diet on gout and inflammation, although this effect is still debated (110). As shown in **Figure 3**, multiple dietary chemicals can act on various aspects of epigenetic regulation in the cells. Resveratrol can affect DNA methylation, histone methylation, histone acetylation, miRs, and histone PTM readers. Genistein can modify levels of DNA methylation, miRs, and histone PTM readers. Sulforaphane affects DNA methylation, histone acetylation, and miRs. The potential for pleiotropic effects, which could be additive, synergistic, or possibly antagonistic, makes the choice of a specific optimal diet a very complex issue. The meta-analysis by Li and colleagues (90) identified families of food which contain dietary factors associated with gout risk. This list should probably be used as the first screen to determine an optimal diet to minimize the negative effects of high uric acid in the blood, and subsequent formation of MSU crystals, thereby avoiding the risk to develop gout and/or reducing inflammatory crises.

## Epigenetic Modifiers, Novel Preventive/Therapeutic Options in Gout?

As the role of epigenetic events in various aspects of gout onset and worsening symptoms become more evident, it opens the door for the development of novel therapeutic strategies. Early studies have demonstrated that drugs acting as inhibitors of histone modifiers (HDACi, HMT or HDM inhibitors) (104, 105) and drugs modifying the DNA methylation status (65) can prevent or reduce gout symptoms at least in certain cell types. One may consider designing a combination of these epigenetic modifying drugs to be provided to gout patients to reduce MSU crystal accumulation (using HDACi), as well as inflammation (through modifying DNA methylation patterns and use of HDACi). One of the main issues to be addressed, as is the case for many other diseases, remains tissue targeting and cell-specificity. Different cell types may react in opposite ways in response to exposure to drugs such as Sodium Butyrate or Vorinostat. Several HDACi, such as valproic acid (VPA), have been tested in patients suffering neurological disorders or cancer with variable levels of success (111). Issues of toxicity on patients for the HDACi drug Trichostatin A limited its therapeutic use (112). A comparable approach to gout prevention or treatment is likely to result in similar adverse effects. As was done in the context of cancer therapy, modulation of DNA methylation using specific DNMT inhibitors (113) might also provide another option to fight gout onset and symptoms. Similarly to what may reduce the efficiency of HDACi-based treatments, targeting and cell-specificity might be a limiting factor.

If one considers prevention or mitigation of gout symptoms through controlling diet, evidence indicate that specific foods should be avoided (3, 90). Red meat, alcohol, fructose, and certain types of seafood were identified as positively correlated with gout and hyperuricemia, where dairy products and soy foods were deemed beneficial. Dairy products should be a source of short-chain FAs and soy food would provide several of the polyphenolic compounds, such as genistein, known to affect DNA methylation (89), miR expression profile (68), histone methylation (68), and histone PTM readers (67). The other polyphenolic compounds common in human diet and displaying potential beneficial effects in reducing inflammation include sulforaphane [in cruciferous plants (88, 91, 92)] resveratrol [in red wine (83, 87)], and curcumin [in turmeric (67, 70, 93)].

Early results from clinical trials on the effect of high ω-3 FA intake on various types of cancers have yielded results showing decreases in symptoms, as well as reduction in inflammation mostly evidenced by lowered NF-κB (114–117). A similar biological response may be expected for gout-suffering patients exposed to a therapeutic regimen of EPA and DHA using a standard dose of 3-9 gram per day (114). A sustained high ω-3 FA daily diet may be considered as a preventive method, but would likely have to be combined with other dietary restrictions.

## Conclusion

**Figure 4:**
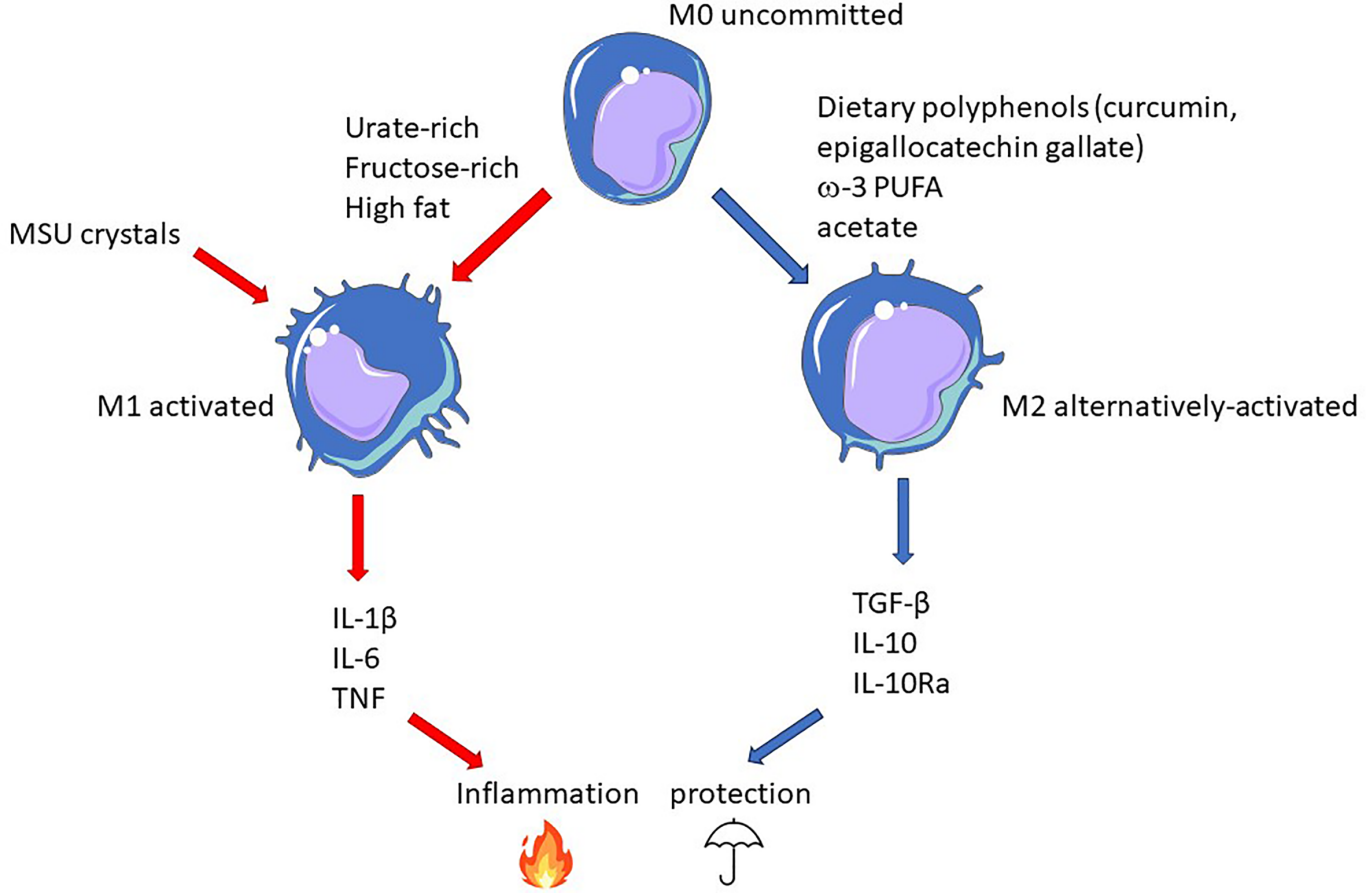
Epigenetic-driven macrophage polarization: an important actionable lever that can be targeted by food-derived inflammatory modifiers. In this model, gout flares result from the conjunction MSU crystals and pro-inflammatory dietary components promoting M1 polarization (red arrows). On the contrary, M2-orienting nutrients favor macrophages that would be more tolerant to crystals and thus confer protection (blue arrows).

Better knowledge of the interactions between diet-derived epigenetic modifiers will be necessary to elaborate an adapted diet, which, combined to pharmacological epigenetic modifiers, should provide long term benefits to gout patients, complementing present therapies (like uricosuric) that act on the short term. As illustrated in **Figure 4**, macrophage polarization appears as an attractive target on which diet can leverage to promote a protective environment towards the risk of MSU crystals-dependent inflammation that may occur in hyperuricemic individuals.

Many additional factors (previous infections, vaccination, physical activities, stress) which are not considered here also have an impact on trained immunity (118) and the activation status of macrophages. While the difficulty to assess and describe the contribution of these non-heritable, environmental factors on inflammation in general and gout in particular, poses a real challenge, the possibility to change them, as opposed to heritable features, makes their identification an exciting field of research and a promising preventive/therapeutic opportunity. With regards to food intake, variations around the Mediterranean diet, rich in fatty acids from fish, high in polyphenols provided by fruits, vegetables, coffee, tea and red wine, shown to reduce age-related decline of cognitive functions (59), might provide a reasonable basis for a compromise aimed at preventing gout’s onset and controlling its progression.

## Author Contributions

All authors listed have made a substantial, direct, and intellectual contribution to the work and approved it for publication.

## Funding

Work in PG’s lab is supported by the Strasbourg’s Interdisciplinary Thematic Instsitute (ITI) for Precision Medicine, TRANSPLANTEX NG, as part of the ITI 2021-2028 program of the University of Strasbourg, CNRS and INSERM, funded by IdEx Unistra (ANR-10-IDEX-0002) and SFRI-STRAT’US (ANR-20-SFRI-0012), the INSERM UMR_S 1109, the University of Strasbourg (IDEX UNISTRA), the European regional development fund (European Union) INTERREG V program (project PERSONALIS) and the MSD-Avenir grant AUTOGEN. Work in PTG’s lab is supported in part by the National Science Foundation, Award Number: 1458952 (Proposal Title: RII Track-1: Gravitational Wave Astronomy and the Appalachian Freshwater Initiative), the Marshall University Genomics Core, Bioinformatics Core and the WV-INBRE grant (P20GM103434) NIH/NIGMS.

## Conflict of Interest

The authors declare that the research was conducted in the absence of any commercial or financial relationships that could be construed as a potential conflict of interest.

## Publisher’s Note

All claims expressed in this article are solely those of the authors and do not necessarily represent those of their affiliated organizations, or those of the publisher, the editors and the reviewers. Any product that may be evaluated in this article, or claim that may be made by its manufacturer, is not guaranteed or endorsed by the publisher.
